# Investigation of the DNA Damage and Oxidative Effect Induced by Venlafaxine in Mouse Brain and Liver Cells

**DOI:** 10.3390/toxics10120737

**Published:** 2022-11-29

**Authors:** Eduardo Madrigal-Bujaidar, Rogelio Paniagua-Pérez, Michael Joshue Rendón-Barrón, José Antonio Morales-González, Eduardo O. Madrigal-Santillán, Isela Álvarez-González

**Affiliations:** 1 Laboratorio de Genética, Escuela Nacional de Ciencias Biológicas, Instituto Politécnico Nacional, Av. Wilfrido Massieu s/n, Zacatenco, Gustavo A. Madero, Ciudad de México 07738, Mexico; 2 Instituto Nacional de Rehabilitación, Servicio de Bioquímica. Av., México-Xochimilco 289, Ciudad de México 14389, Mexico; 3 Laboratorio de Medicina de la Conservación, Escuela Superior de Medicina, Instituto Politécnico Nacional, Plan de San Luis y Díaz Mirón s/n, Casco de Santo Tomás, Ciudad de México 11340, Mexico

**Keywords:** venlafaxine, genotoxicity, molecular oxidation, mouse brain and liver

## Abstract

Venlafaxine is an antidepressant used worldwide. Therefore, studies to confirm its safe use are mandatory. This report evaluated the drug DNA damage capacity in the brain and liver of ICR mice, and its oxidative effect on DNA, lipids, and proteins, as well as the amount of nitrites, also in the brain and liver. Determinations were made at 2, 6, 12, and 24 h post-treatment, excluding DNA oxidation that was observed at 2 h. The tested doses of venlafaxine were 5, 50, and 250 mg/kg. The results showed DNA damage in the brain with the two more elevated doses of venlafaxine at 2 and 6 h post-treatment and also at 12 h in the liver. The comet assay plus the FPG enzyme showed DNA damage in both organs with all doses. The two high doses increased lipoperoxidation in the two tissues from 6 to 12 h post-administration. Protein oxidation increased with the three doses, mainly from 2 to 12 h, and nitrite content was elevated only with the high dose in the liver. The drug was found to affect both tissues, although it was more pronounced in the liver. Interestingly, DNA oxidative damage was observed even with a dose that corresponds to the therapeutic range. The clinical relevance of these findings awaits further investigations.

## 1. Introduction

Venlafaxine was the first synthesized antidepressant drug belonging to the group of inhibitors of serotonin and noradrenaline recapture drugs. It is a racemic compound named 1-[2-dimethylamine-1-(4-methoxyphenil)ethyl]ciclohexan-1-ol [1]. The chemical is metabolized in the liver by the action of the cytochrome P450 enzyme to the active metabolite O-desmethylvenlafaxine through the processes of conjugation and oxidation to a lesser extent. Venlafaxine and its main metabolite exhibit linear kinetics with an elimination half-life of 5 ± 2 h and 11 ± 2 h, respectively. Regarding the clinical use of this antidepressant, the maximum recommended dose is 375 mg/day [2].

The drug has been recommended in patients with major depression but also for the improvement of other diseases, including generalized anxiety disorders, panic attacks, social phobia, and neuropathic pain, among its various uses [3]. With respect to its toxicity, it has been observed that the drug may cause various collateral organic damage, such as neurological and cardiovascular effects, as well as rhabdomiolisis, interstitial pneumonitis, and prostatism. In addition, a controlled study performed in rats also demonstrated a number of histopathological damages in the liver, kidney, and stomach [4]. With respect to the genotoxic field, a report by Brambilla in 2009 [5] mentioned that no damage had been shown by venlafaxine in various in vitro and in vivo studies; however, Lacaze et al. [6] reported a study conducted in hemocytes of blue mussel (*Mytilus edulis*), where increased phagocytosis activity was found by exposure to venlafaxine, as well as moderate DNA damage determined with the comet assay. Another report using cultured human lymphocytes demonstrated a significant increase in chromosomal aberrations, mainly chromatid breaks and sister chromatid unions, as well as micronuclei in binuclear cells [7]. Finally, Madrigal-Bujaidar et al. [8], using the micronucleus test in mice, determined significant genotoxic damage, as well as bone marrow proliferation decrease, by registering the number of immature and mature erythrocytes. Moreover, in another area, a study conducted in rat hepatocytes demonstrated that antidepressants induced lipid peroxidation, cellular GSH content depletion, and elevation of the GSSG level and consequently increased the formation of ROS [9]. These findings are of high significance in light of the well-known knowledge that oxidative stress is a significant etiological factor that causes lipid, protein, and DNA damage and because these alterations may be associated with a number of genetic damages that may trigger the development of numerous diseases, including cancer [10].

Therefore, the design of the present report was based on the following points: initially, because depression is a public health concern that constitutes one of the most common mental diseases worldwide and because it is also the second leading disorder that causes years of living with disability, a problem with high prevalence among people of working ages, depression results in a strong economic burden [11]. Moreover, because of the mentioned importance, the use of antidepressant drugs has been highly relevant to combat the disease and, in this form, to improve the socioeconomic burden. However, some of the antidepressant drugs used have been reported to possess variable genotoxic effects, including venlafaxine. Therefore, based on the previous information, the first aim of the present report was to evaluate the genotoxic potential of venlafaxine with a different approach, which includes the use of the comet assay in an in vivo kinetic time strategy design in mouse brain and liver cells. For this purpose, we studied DNA damage at 2, 6, 12, and 24 h post-administration of the drug; moreover, the oxidative capacity of venlafaxine was determined in the same organs. In this case, the DNA oxidative potential was examined after 2 h of administration, and the lipid and protein oxidation, as well as the nitrite content, was determined at 2, 6, 12, and 24 h.

## 2. Materials and Methods

### 2.1. Chemicals and Animals

Venlafaxine was obtained as the usually prescribed antidepressant (Effexor, Pfizer, México City, México), CAS number 136434-34-9, and molecular formula C17H27NO2. The following substances were obtained from Sigma Chemicals (St Louis, MO, USA): Triton X-100, dimethyl sulfoxide (DMSO), methyl metanesulfonate (MMS) sodium chloride, tris, normal melting point agarose (NMPA), low melting point agarose (LMPA), calcium, magnesium-free phosphate-buffered saline (PBS), ethidium bromide (EB), trypan blue solution, N-lauroyl-sarcosine (sodium salt), HEPES, bovine serum albumin (BSA), Bradford reagent, thiobarbituric acid (TBA), trichloroacetic acid (TCA), 2,4-dinitrophenylhydrazine (DNPH), guanidine, sulfanilamide, N-1-(naftil) etilendiamine dichloride, and the enzyme formamidopyrimidine-DNA glycosylase (FPG). In addition, potassium hydroxide, potassium chloride, sodium hydroxide, EDTA, ethanol, ethyl acetate, formic acid, methanol, and hydrochloric acid (HCl) were purchased from Baker (Phillipsburg NJ, USA).

For the assay, we used 100 six-week-old male mice (ICR) with a mean weight of 24 g (Harlan Laboratory, Mexico City, Mexico). Five animals per cage and per experimental group were placed in polycarbonate cages at 24 °C, 12 h dark–light cycles, and 50% relative humidity, and the animals had free access to water and food (Rodent Lab Chow 5001, Purina). The experiment was approved by the Bioethics Committee of the Hidalgo State Autonomous University (11 January 2021. Pachuca, Hidalgo, México), and it was started after a week of mouse conditioning in the Genetics Animal Facility of the Laboratory of Genetics, according to the previously mentioned conditions. Moreover, the animal management of the present study followed the international guidelines and regulations described in ARRIVE guidelines 2.0 [12].

With respect to the single cell gel electrophoresis assay (SCGEA), we used the next groups of animals, each of them with 25 mice: a control group of mice was intragastrically (IG) given purified water, a positive control group was intraperitoneally (IP) injected with 150 mg/kg MMS, and three additional groups were IG administered venlafaxine with 5, 50, and 250 mg/kg, respectively. The drug was dissolved in purified water (0.5, 5, and 25 mg/mL) to finally administer the corresponding doses. The last tested dose was approximately 80% of the LD_50_ previously determined in our laboratory by the IG path (using the method of Lorke [13]); besides, the lower dose agreed with the high quantity prescribed for the daily therapeutic drug in humans [2]. Concerning the oxidative tests, in the same mice used for the comet assay, we applied the same chemicals and used the same routes and doses as already described for the negative control, positive control, and venlafaxine-treated groups. Mice were euthanized by cervical dislocation, and determinations of each oxidative and genotoxic parameter were conducted at 2, 6, 12, and 24 h post-administration (five animals per group), with the exception of the modified comet assay with the FPG enzyme. In this case, the evaluation was made at 2 h only because the results observed with the classic SCGEA suggested that DNA damage was rapidly induced by the drug.

### 2.2. SCGEA Standard Method

The comet assay is based on the migration of negatively charged, fragmented DNA strands when subjected to an electrical field. The procedure includes the preparation of a cell suspension layered onto slides, the cell lysis in a high salt solution, the DNA unwinds, and its electrophoresis under alkali conditions before being stained with a fluorescent dye. In the following lines, we describe the procedure made in the present report. Initially, we obtained the whole brain and a fragment of the right liver lobule from each mouse. With respect to the brain, approximately 4 mm of the tissue was placed in 500 µL of cold PBS and several times hit with a syringe rod to eventually place 40 µL of the cell suspension in a tube on ice. The liver tissue was disjoined with scissors, the clumps were detached, and the cells were placed in PBS at 4 °C. The method of the SCGEA followed the guidelines published by OECD 489 [14]. We used approximately 10,000 cells/mL in each tested sample with the viability of no less than 80% according to the trypan blue staining method.

Fully frosted slides were initially coated with three layers of agarose. The first layer consisted of 0.12 mL of 1% NMPA made in PBS. Above this layer of agarose, we aggregated a second sheet formed by 0.075 mL of 1% LMPA prepared in PBS plus 0.02 mL of brain or liver mass, and the last coat was formed by 0.075 mL of 1% LMPA. From each animal, three slides protected from light were made per treatment/exposure/time and stored for 24 h at 4 °C in lysis solution (NaCl 2.5 M EDTA 100 mM, tris 10 mM, 1% sodium sarcosinate, 1% Triton X-100, and 10% DMSO at pH 10). Slides were then placed in an electrophoresis chamber containing 300 mM NaOH plus 1 mM EDTA at pH > 13 and 4 °C for 20 min before the electrophoresis (25 V, 250 mA, and pH > 13 for 20 min). Then, the slides were washed with Tris (0.4 M, pH 7.5) for 5 min. The stain was made with EB (25 µg/mL), and finally, the length-to-width relationship was determined in 100 nucleoids per individual/treatment/time using an epifluorescence microscope (Axioscope, Carl Zeiss, Jena, Germany) equipped with emission and excitation filters of 488 and 565 nm, respectively. In addition, the microscope was adjusted to the image analyzer Image-Pro Plus (Media Cybernetics, Silver Spring, MD, USA). This type of comet measurement includes two steps: tail length measurement and nucleoid diameter measurement [15,16].

### 2.3. SCGEA with the FPG Enzyme

To evaluate the capacity of venlafaxine to oxidize the DNA molecule, we applied the comet assay plus the addition of the FPG enzyme. Slides parallel to those prepared for the previous assay were used. In this second comet method, however, after cells went through the lysis phase, they were laundered with the enzyme buffer (3 times for 5 min each). The enzyme buffer was composed of 40 mM HEPES, 0.1 M KCl, EDTA 0.5 mM, and albumin bovine serum 0.2 mg/mL, pH 8.0. Then, 10 µg of the FPG enzyme was diluted in 2 mL of the buffer solution to produce a stock solution of 5 µg/mL, and 10 µL of this solution was aggregated to 40 µL of the buffer solution to obtain an FPG concentration of 1 µg/mL. From this preparation, 50 µL of the enzyme was added to each slide; one of these (per mouse) was exposed to 50 µL of the FPG buffer after 2 h of venlafaxine administration. In the next step, all slides were put in a wet chamber for 45 min at 37 °C. After that, the slides were located at 4 °C for 5 min, the coverslips were detached, and DNA denaturation was made with a solution of 300 mM NaOH plus 1 mM EDTA at pH 13 for 40 min; at the end, electrophoresis was accomplished at 25 V, 300 mA, and pH > 13 for 30 min [17]. After that, the scoring and statistical analysis were performed as described above for the classic SCGEA method. To obtain the DNA oxidation, the values which were determined with the enzyme buffer were subtracted from the results obtained with the FPG solution.

### 2.4. Total Protein Evaluation

The indicated evaluation was made according to the method of Bradford [18]. The brain and the liver were homogenized in cold PBS (1:10 *w*/*v*), and 0.1 mL of the homogenate from each sample was centrifuged at 13,500× *g* for 10 min. Then, to 0.01 mL of the obtained supernatant, we added 2.5 mL of Bradford’s reagent plus 0.09 mL of deionized water. The mixture was agitated for 5 min, and the absorbances of the samples were measured at 595 nm using a blank made with 0.1 mL of deionized water plus 2.5 mL of Bradford’s reagent. The results were interpolated in a bovine serum albumin standard curve (from 0.1 to 1.0 mg/mL) and expressed as mg protein/g tissue.

### 2.5. Evaluation of Lipoperoxidation

Malondialdehyde (MDA) is one of the final products of polyunsaturated fatty acids peroxidation in cells. MDA level is a commonly known marker of oxidative stress and antioxidant status. This determination was made according to the method of Buege and Aust [19]. Initially, the brain and the liver were homogenized 1:10 (*w*/*v*) in cold PBS. After that, in a test tube per sample, we added 0.5 mL of the homogenate from each tissue plus 2 mL of the reaction mixture (TCA-TBA-HCl) at 15% *w*/*v*, 0.375% *w*/*v*, and 0.25 N, respectively. The samples were boiled for 15 min, cooled for 10 min in an ice bath, and centrifuged at 2600× *g* for 10 min. The absorbances of the supernatant were spectrophotometrically measured (Spectronic 20, Genesys^TM^, Baton Rouge, LA, USA) at 532 nm, utilizing as a blank 0.5 mL of PBS and 2.5 mL of the reaction mixture. The concentration of MDA was estimated by using an extinction coefficient of 1.56 × 10^5^ M^−1^ cm^−1^, and the results were shown as nmol MDA/mg protein.

### 2.6. Evaluation of Oxidized Proteins

Protein oxidation is mainly caused by reactive oxygen and nitrogen species, is thought to play a major role in various oxidative processes within the cells, and is implicated in the development of many human diseases. In this determination, we followed the method of Levine et al. [20] to determine the reactive carbonyl content. The liver and the brain of each mouse were homogenized in PBS 1:10 (*w*/*v*). Later, we added 0.2 mL of the tissue homogenate plus 0.5 mL of DNPH (10 mM in HCl 2 mM). The blend remained at rest in the dark at room temperature for 1 h, and the generated hydrazones were precipitated with 0.5 mL of 20% TCA. Each sample was centrifuged three times at 13,500× *g* for 10 min, and each time, the suspension was washed with 1 mL of ethyl acetate–ethanol 1:1. The pellet was resuspended in 1 ml of hydrochlorate guanidine 6 M, incubated at 37 °C for 15 min, and centrifuged at 11,000× *g* for 10 min. The blank was incubated at 37 °C with 0.5 mL of HCl 2 M without DNPH. The amount of carbonyl was spectrophotometrically registered in a range from 350 to 375 nm (Spectronic 20, Genesys^TM^, Baton Rouge, LA, USA), and its concentration was calculated by using 22,000 M^−1^cm^−1^ as the coefficient of molar absorbance. The results were expressed as nmol of CO/mg protein.

### 2.7. Nitrite Determination

Nitrite may generate NO and, in this form, participates in numerous biological pathways, including a number of cell injuries and diseases. Here we show the nitrite determination. The brain and the liver were washed with PBS 0.01 M at 4 °C, pH 7.4. Then, a homogenate was performed with each sample in cold PBS (1:4 *w*/*v*). The mixture was centrifuged at 2600× *g* for 20 min, and the supernatant was treated with the Griess reactive to obtain the nitrite concentration [21]. The procedure consisted of the mixing of 0.1 mL of the supernatant plus 0.6 mL of distilled water and 0.3 mL of the Griess reagent. The absorbance of such mix was registered at 540 nm (Spectronic 20, Genesys^TM^, Baton Rouge, LA, USA). As a standard, we used 0.1 mM NaNO_2_ in an interval from 0.9 µM to 10 μM. The obtained results were shown as nmol of nitrite/g of tissue.

### 2.8. Statistical Analysis

The results were represented as the mean values plus/minus standard deviation (SDM). The results were statistically managed with a two-way ANOVA and with a post hoc Tukey test. For this aim, we utilized the Program Sigma Stat version 3.5 (Systat Software, Inc., Palo Alto, CA, USA).

## 3. Results

### 3.1. Standard and FPG-Modified Single Cell Gel Electrophoresis Assays

**Figure 1:**
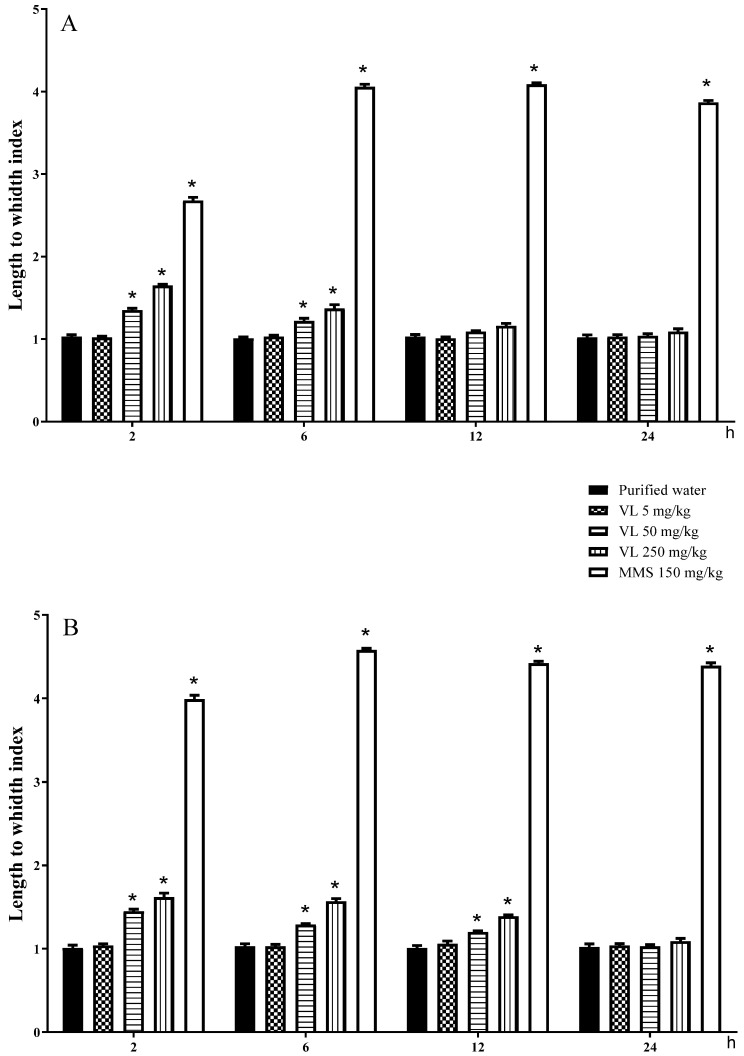
DNA damage induced by venlafaxine (VL) in the brain of mouse (**A**) and in mouse hepatic tissue (**B**). MMS = methyl methanesulfonate. Each bar represents the average ± SDM determined in 100 nucleoids per mouse. Five mice per dose. * Shows statistically significant difference in comparison with the control level. Two-way ANOVA and Tukey tests (*p* ≤ 0.05).

The results determined with the standard comet assay in the brain are shown in Figure 1A. Low and uniform DNA damage levels were found in the control mice throughout the experimental period, with a mean length-to-width index value of 1.02 ± 0.009, which contrasts with the significant DNA damage exerted by MMS throughout the whole schedule, with a mean damage elevation of approximately three and a half times over the control level. With respect to the effect of venlafaxine, the low dose gave rise to values similar to those observed in the control group; however, the intermediate dose of the drug produced significant DNA damage at 2 and 6 h of exposure, while the high tested dose (250 mg/kg) also induced more evident damage at 2 and 6 h post-exposure. At 2 h, the high dose reached an approximately 60% increase over the control level. A damage decline related to the time elapsed in the assay was observed with the two higher doses, surely related to the repair and detoxification processes.

With respect to the liver (Figure 1B), the mean increase produced with MMS was more than four times the mean control level, and with respect to the effect of venlafaxine, we newly determined no DNA damage with the low dose, although significant damage was found with the two high doses at 2, 6, and 12 h of exposure. The damage was more elevated than that in brain cells. Again, a decline in the effect related to the exposure time was observed.

**Figure 2:**
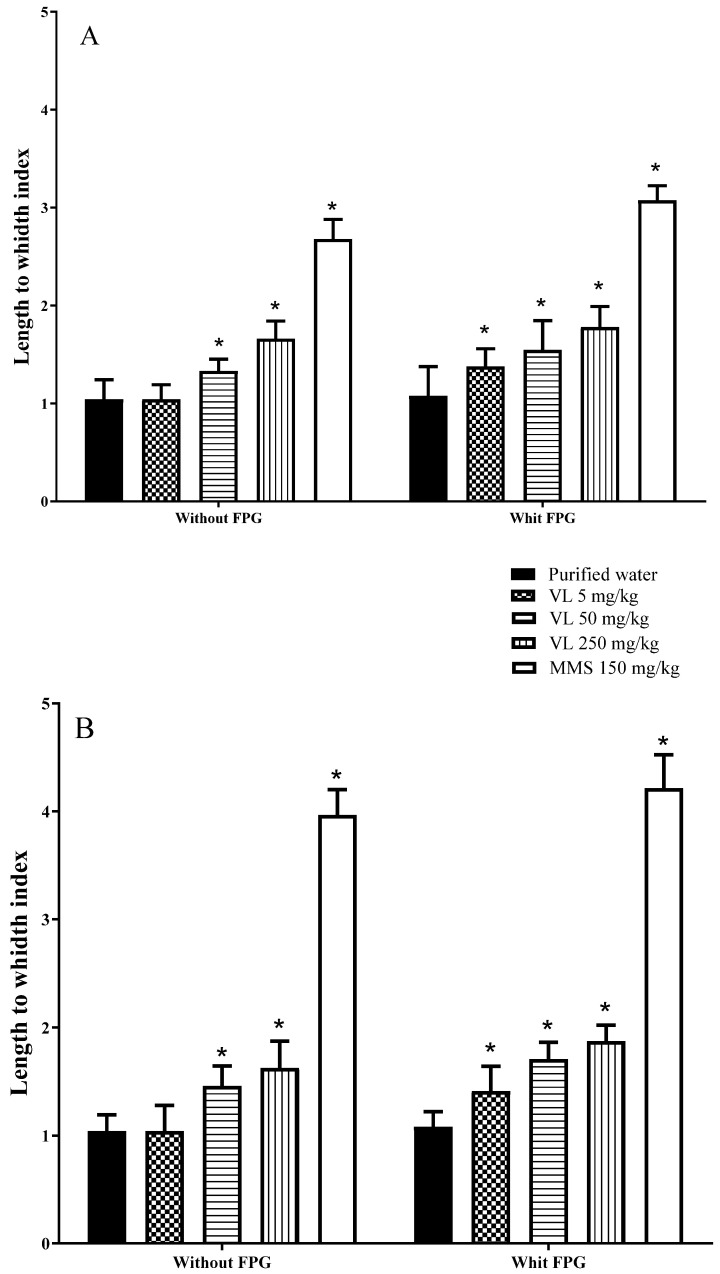
DNA damage induced at 2 h of venlafaxine (VL) exposure without and with the FPG enzyme. In the brain of mouse (**A**) and in mouse hepatic tissue (**B**). MMS = methyl methanesulfonate. Each bar represents the mean ± SDM obtained in 100 nucleoids per mouse. Five mice per dose. * Shows statistically significant difference in comparison with the control level. Two-way ANOVA and Tukey tests (*p* ≤ 0.05).

Figure 2A shows the results obtained in the brain with the comet assay plus the FPG enzyme. After 2 h of exposure, DNA damage related to the oxidation process was observed in the treatments with MMS and venlafaxine along the studied schedule. The mean increase in the drug’s DNA oxidative damage corresponded to 16.4% over the mean result obtained with the standard comet test. Interestingly, in the comet-FPG assay, it was noted that the low tested dose (5 mg/kg) produced a significant DNA-breaking effect related to the oxidation process, a different finding from that observed in the standard comet assay. The results obtained in the liver with the comet-FPG test (Figure 2B) were similar to those described in the brain: an elevation of DNA damage by MMS and venlafaxine in comparison with data obtained with the application of the standard comet assay, giving rise to a mean increase of 21% effect by the antidepressant, as well as a significant increase with its low tested dose.

### 3.2. Biomolecule Oxidation and Nitrite Content

**Figure 3:**
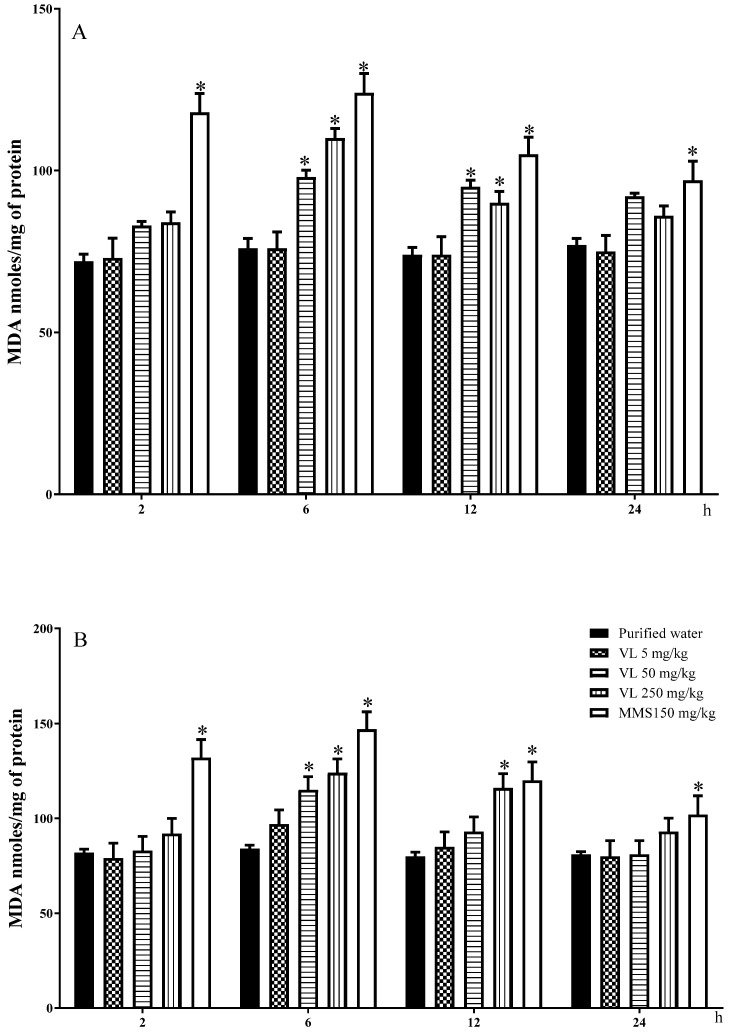
Lipid peroxidation induced by venlafaxine (VL) measured with the malondialdehyde content (MDA) in the brain of mouse (**A**) and in mouse hepatic tissue (**B**). MMS = methyl methane- sulfonate. Each bar represents the mean ± SDM obtained in 5 independent determinations per dose. * Shows statistically significant difference in comparison with the control level. Two-way ANOVA and Tukey tests (*p* ≤ 0.05).

Information about brain lipoperoxidation is shown in Figure 3A. The MDA values observed in the control group were similarly maintained throughout the evaluated times, with a mean value of 74.75 ± 2.21 nmol/mg of protein, while the values obtained by the effect of MMS were over 90 nmol/mg of protein at all the evaluated times. Regarding the low dose of venlafaxine, no lipid oxidation was detected in the brain; however, at 6 and 12 h, the effect produced with 50 and 250 mg/kg was significant in comparison with the control level, with a decline at 24 h.

In the case of liver peroxidation (Figure 3B), a similar pattern to that described before was observed with respect to the control and the MMS-treated mice, and with respect to venlafaxine, the results showed no effect with the low tested dose and a significant effect with 50 and 250 mg/kg of the drug at 6 h of exposure and with the high dose at 12 h.

**Figure 4:**
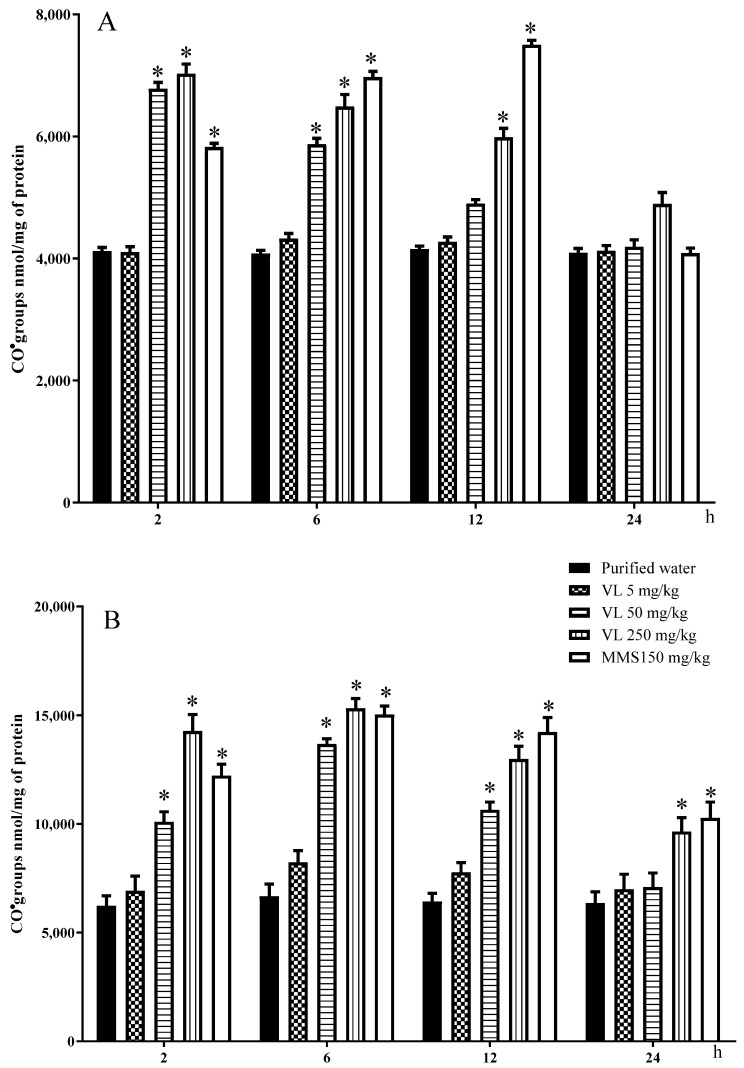
Effect of venlafaxine (VL) on the reactive carbonyl content (CO^●^) in the brain of mouse (**A**) and in mouse hepatic tissue (**B**). MMS = methyl methanesulfonate. Each bar represents the mean ± SDM obtained in 5 independent determinations per dose. * Shows statistically significant difference in comparison with the control level. Two-way ANOVA and Tukey tests (*p* ≤ 0.05).

Protein oxidation through reactive carbonyl quantification is presented in Figure 4A,B in regard to the brain and liver, respectively. In the brain, the control values were concordant throughout the study, with a mean value of 4111 ± 31 nmol CO/mg of protein, and the MMS-treated cells showed a stronger effect from 2 to 12 h of exposure, with a mean increase of 64% over the mean control level. Concerning the venlafaxine effect, the low tested dose showed no difference in comparison with the control level throughout the assay; however, the two high doses were significantly elevated at 2 and 6 h, and the high dose was also significant at 12 h. At 24 h, none was significant. In the liver, the control and the MMS-treated groups showed higher values than in the brain, although the general behavior was similar. In relation to 50 mg/kg of venlafaxine, the results showed no significant elevation of oxidized proteins along the observed schedule, although its administration induced a certain increase, the two high doses were significant from 2 to 12 h post-administration, and the high dose (250 mg/kg) was also significant at 24 h. The effect of this dose of the drug was somewhat similar to the effect induced by MMS. In the period from 2 to 12 h, the mean increase in venlafaxine (50 mg/kg) was 78% higher than the control level, and 250 mg/kg was 120% higher, indicating a stronger effect in this organ with respect to the brain.

**Figure 5:**
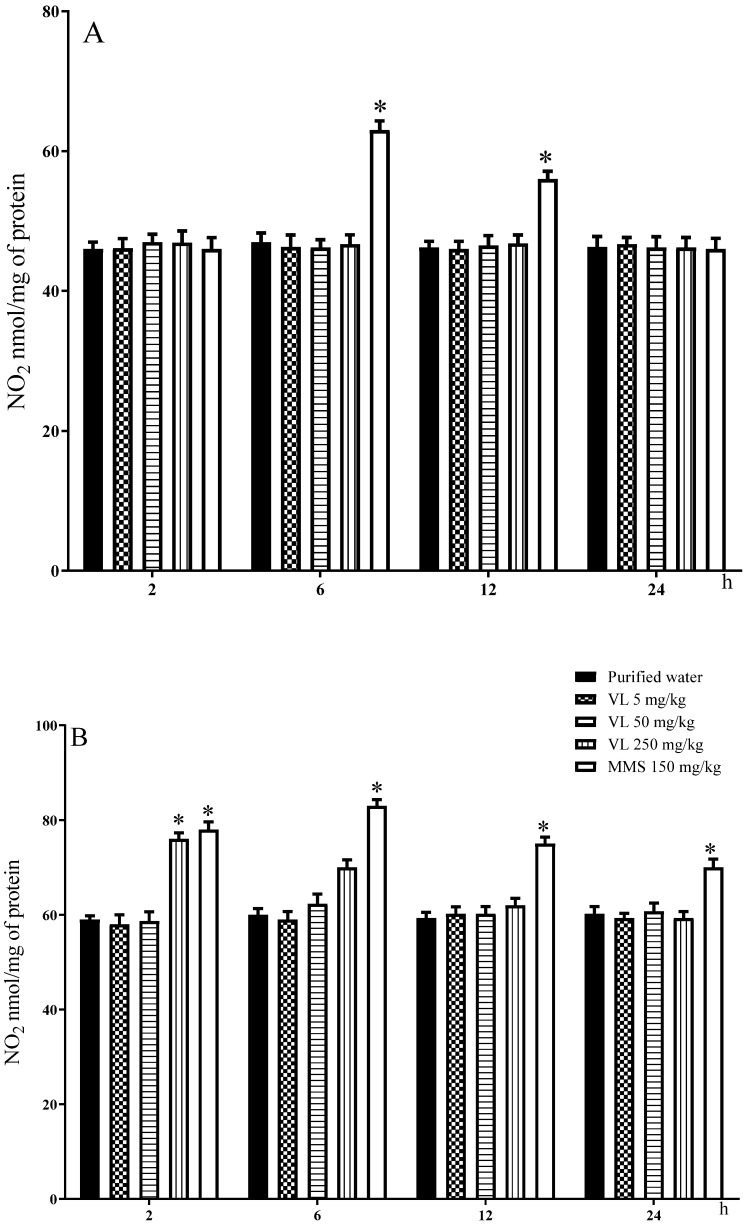
Effect of venlafaxine (VL) on the nitrite content (NO_2_) in the brain of mouse (**A**) and in mouse hepatic tissue (**B**). MMS = methyl methanesulfonate. Each bar represents the average ± SDM found in 5 separated observations per dose. * Shows statistically significant difference in comparison with the control level. Two-way ANOVA and Tukey tests (*p* ≤ 0.05).

Figure 5A presents the results found with respect to the content of nitrites in the brain. Control cells had homogeneous values throughout the study, with a mean of 46.37 ± 0.43 nmol of NO_2_/g of tissue, and the MMS-treated group showed a significant increase at 6 and 12 h of its administration. With respect to venlafaxine, in this determination, we detected no effect along the studied schedule. However, in liver tissue (Figure 5B), the nitrite content induced at 2 h of venlafaxine exposure (250 mg/kg) was statistically elevated compared to the control level, suggesting more sensitivity to nitrite induction in this organ. MMS was statistically significant from 2 to 24 h.

## 4. Discussion

The etiology of major depressive disorder is believed to be multifactorial, including the interaction of social, psychological, and biological aspects, and its prevalence varies in relation to the country’s culture. For example, in the Czech Republic, a 12-month prevalence of 0.3% was reported, which is highly different from the observed in other countries, for example, 5.2% in West Germany, 4.5% in Mexico, and 10% in the USA [22,23].

Depression may be treated with psychotherapy, neuromodulation technology, and drug therapy. In general, mild depression is usually treated with a psychosocial approach, and drug therapy seems more effective in moderate to severe cases of the disease [24].

In the last ten years, hot research topics have notably increased in number of publications, including those related to depression management in primary care, interventions to prevent the disease, studies on the pathogenesis of depression, its comorbidities, and antidepressant treatment [25]. Regarding the last point, the development of new antidepressants has increased in recent years, and presently, we count nine different types of drugs approved and used across the world, including those with psychopharmacological mechanisms of effect based on serotonin and norepinephrine reuptake inhibitors, which correspond to the class of venlafaxine.

One of the topics with low to moderate investigation corresponds to the safe use of antidepressants, including the genotoxic and cytotoxic fields of knowledge; however, the published information is sufficient to make clear that a number of drugs to treat depression may have DNA-damaging potential. Such studies have been made, for example, in imipramine, desipramine, milnacipram, trazodone, citalopram, sertraline, and duloxetine, among other drugs [26,27,28,29,30]. 

Information about alterations to the DNA molecule by venlafaxine is limited. Lacaze et al. [5] reported moderate DNA damage in the hemocytes of blue mussels. Sołek et al. [31] determined the promotion of a telomerase-focused DNA damage response in mouse spermatogenic cells studied in vitro; however, Saleem et al. [32] found no DNA damage in rat spermatozoids studied in vivo. On the other hand, chromosome studies, mainly performed with the micronucleus test, have been positively consistent for venlafaxine genotoxicity; in this area, reports have been made in mice in vitro using spermatogenic cells and in mice in vivo using an acute and a subchronic approach [7,31]. In the present research, we found significant DNA damage induced with 50 and 250 mg/kg of venlafaxine at 2 and 6 h postexposure in brain cells, as well as in liver cells, although in this last tissue, the effect was also observed at 12 h of exposure. In addition, significant DNA damage was found when the oxidation of the molecule was considered; thus, in mice examined with the comet-FPG test, the three tested doses of venlafaxine were superior to the result obtained in the same groups without the enzyme. It was particularly relevant that the low tested dose (5 mg/kg), which belongs to the higher therapeutic drug prescription range, was able to damage DNA. The FPG enzyme mainly determines purines that have been oxidized, as well as formamidopyrimidines (ring-opened adenine or guanine) and ring-opened N7 guanine adducts, products that enhance the sensitivity of the comet assay by converting damaged bases to breaks [33].

The present findings about venlafaxine DNA damage potential are relevant in light of the described relationship of cellular DNA alterations with the etiology and progression of many different types of human disorders and diseases, including neurodegenerative conditions, skeletal damage, mental disorders, hematological abnormalities, and cancer development [34,35]. In major depression, in particular, the contribution of genetics has been suggested to be between 40 and 50%, an influence that has been related to polygenic effects, epigenetic factors, or the presence of single nucleotide polymorphisms [25,36].

In our present assay, in addition to DNA oxidation, the oxidative effect of the evaluated drug was also found in lipids and proteins of the studied tissues. With respect to proteins, the two high doses elevated the liver oxidation from 2 to 12 h post-treatment and in brain cells from 2 to 6 h; also, brain lipoperoxidation was increased with the two high doses from 6 to 12 h, and in liver cells, the increase was found with the high dose from 6 to 12 h post-administration. Again, as observed in the case of DNA, the protein and lipid oxidation decreased toward 24 h post-administration.

Our present study showed moderate nitrosative effects with the administration of 250 mg/kg venlafaxine only in the liver, a result probably related to the intrinsic properties of the drug or the experimental conditions used. However, exposure to venlafaxine demonstrated clear lipid oxidative damage by the measurement of MDA, a molecule produced by polyunsaturated fatty acids and one of the best investigated products of lipid peroxidation. MDA is a relevant molecule because of its various interactions; for example, the compound may affect proteins and DNA independently or may produce protein/DNA crosslinking into the mentioned molecules or between the two molecules, which may cause extensive biochemical damage in the cells when the lipid peroxidation rate overwhelms its repair capacity; in this form, apoptosis and necrosis may be induced as well as the development of a number of pathological states, such as cancer, diabetes, cardiovascular, neurological and inflammatory diseases, and the acceleration of the aging process [37,38]. In the case of proteins, their radical-mediated damage may be initiated by electron leakage, metal-ion-dependent reactions, and oxidation of lipids and sugars. A number of oxidized proteins are poorly handled by cells and may then accumulate and participate in aging and pathologies such as diabetes, atherosclerosis, or neurodegenerative diseases. Proteins are major targets for oxidation reactions because of their rapid reaction rates with oxidants and their high abundance in cells, extracellular tissue, and body fluids; moreover, as shown in the present study, a correlation between MDA and the number of protein modifications has been suggested [39].

The observed biomolecular oxidative damage agrees with other studies on the matter. For example, Ahmadian et al. [8] studied collagenase-perfused rat hepatocytes treated with venlafaxine and demonstrated the induction of ROS followed by lipid peroxidation, cellular GSH content depletion, elevated GSSG levels, and loss of lysosomal membrane integrity, among other disturbances. However, studies about the oxidative/antioxidative properties of antidepressants have been inconclusive for a number of years. Regarding venlafaxine, other authors have found antagonizing effects on oxidative stress, enhancing antioxidant defense mechanisms, or regulating the activity of epigenetic genes in this area; for example, mouse hippocampal damage by alterations in lipid peroxidation, glutathione, or GST activity was improved by the drug, as well as a number of oxidative parameters that caused cisplatin nephrotoxicity or oxidative-induced rat arthritis [40,41,42,43]. Therefore, in light of the present knowledge, it seems that the pro-oxidant or antioxidant activity of antidepressants can be related to the involved specific drug, the examined tissue, the dose used, and the duration of the assay.

In conclusion, in the present report, we followed the effect of three single doses of venlafaxine through a 24 h period and demonstrated the DNA damaging potential of the drug in mouse brain and liver cells in the initial and middle periods of the examined schedule (2–12 h post-administration) with nontherapeutic doses and its DNA oxidative effect even with a low therapeutic dose at 2 h post-administration. The report also demonstrated the oxidative effect of venlafaxine on lipids and proteins in mouse brain and liver cells at nontherapeutic doses throughout the assay. In addition, it was shown that the molecular effect starts as soon as 2 h and diminishes in all molecular targets toward 24 h post-administration. Our study therefore suggests caution with the therapeutic use of venlafaxine.

## Data Availability

Data are available from the corresponding author upon request.
